# Translation-invariant optical neural network for image classification

**DOI:** 10.1038/s41598-022-22291-0

**Published:** 2022-10-14

**Authors:** Hoda Sadeghzadeh, Somayyeh Koohi

**Affiliations:** Department of Computer Engineering, Sharif University of Technology, Tehran, Iran

**Keywords:** Engineering, Mathematics and computing, Optics and photonics

## Abstract

The classification performance of all-optical Convolutional Neural Networks (CNNs) is greatly influenced by components’ misalignment and translation of input images in the practical applications. In this paper, we propose a free-space all-optical CNN (named Trans-ONN) which accurately classifies translated images in the horizontal, vertical, or diagonal directions. Trans-ONN takes advantages of an optical motion pooling layer which provides the translation invariance property by implementing different optical masks in the Fourier plane for classifying translated test images. Moreover, to enhance the translation invariance property, global average pooling (GAP) is utilized in the Trans-ONN structure, rather than fully connected layers. The comparative studies confirm that taking advantage of vertical and horizontal masks along GAP operation provide the best translation invariance property, compared to the alternative network models, for classifying horizontally and vertically shifted test images up to 50 pixel shifts of Kaggle Cats and Dogs, CIFAR-10, and MNIST datasets, respectively. Also, adopting the diagonal mask along GAP operation achieves the best classification accuracy for classifying translated test images in the diagonal direction for large number of pixel shifts (i.e. more than 30 pixel shifts). It is worth mentioning that the proposed translation invariant networks are capable of classifying the translated test images not included in the training procedure.

## Introduction

It today’s world, machine learning technology is dramatically used in various applications, such as speech recognition, language translation, and decision making to name but a few^1^. Of so great significance are CNNs that it would not be exaggerating to say that the performance of computer vision applications, specifically image classification, are greatly influenced by them^2,3^. However, CNNs operate in the order of giga multiplication-addition operations^4^ which consequently cause different drawbacks, including high power consumption and high memory requirements^5^, to name but a handful. Although graphical processing units (GPUs) and tensor processing units (TPUs) enhance computational performance^5^, high computation time as well as high energy consumption are challenging issues in real-time inference^6^.

Recently, researchers have been motivated to explore substitute method to overcome electrical bottlenecks^7^. Fascinating properties of optical processing, namely high propagation speed of 300,000 km per second, massive parallelism, scalability, power efficiency, and great anti-interference, can drastically solve electrical implementation problems^7,8^. In this regard, many researches have proposed optical deep learning systems^9–13^, as well as new optical implementations of CNNs (OPCNNs)^3,5,6,10,14–18^.

Convolution as a most time-consuming operation can be realized by the common 4f optical correlator in free-space optics^3,5,6,14,15,17,18^. Furthermore, some researches proposed optical nonlinearity^3,18–21^, while few of them proposed optical pooling operation as well^3,15,18^. The authors in^3^ proposed OP-AlexNet architecture which has five convolutional layers and three fully connected (FC) layers. Array of 4f optical correlators is considered as the optical convolutional layer, saturable absorption is considered as the optical nonlinearity unit, and finally, pinhole and Gaussian filter masks perform optical average pooling and optical motion pooling operations, respectively. Notwithstanding the fact that significant progress occurred in implementing different OPCNNs, the classification performance of OPCNNs are influenced by components’ misalignment and translation of input image data in practical applications, yet^17^. Moreover, all recent studies addressing optical neural networks^5,16,17,22^ have pointed out that the accuracy of their experiments are less than those of simulation results due to the presence of misalignment of optical components in reality. As addressed in^17^, improving translation invariance property of the network helps the experimental setup to not being sensitive to the alignment errors, and so, alignment sensitivity reduction is a valuable achievement for the optical setup. Therefore, there is still the requirement of extra methods and components to overcome aforementioned position variation problems for implementing OPCNNs.

Based on above discussion, to overcome misalignment errors in optical implementation, designing translation invariant architecture is of great interest, as discussed as follows. Although many studies address invariance problem of electrical CNNs^23^, a few researches have proposed optical solutions ensuring translation invariance property of OPCNNs. The authors in^24^ proposed a translation invariant VanderLugt correlator by the usage of dc-suppressed holographic filter. In other research^25^, the authors proposed hybrid opto-electronic correlator (HOC) with spatial light modulators (SLMs), detector arrays, and field programmable gate arrays (FPGAs) for shift invariant target recognition. Due to the speed limitation caused by the serial communication between the devices within the HOC, the authors in^26^ proposed a system called Integrated Graphic Processing Unit (IGPU) to increase the processing speed of HOC. However, it is obvious that the usage of application-specific integrated circuits (ASIC) limits the system scalability for big data processing.

Recently, the authors in^15^ proposed an optronic CNN, where convolution of which is performed by a 4f optical correlator and the extracted features pass through CMOS camera. While the optronic CNN utilizes electrical shifted Rectified Linear Unit (ReLU), the optical convolution layer is followed by an optical demagnification system which acts as a strided convolution block performing the pooling operation. Because of the misalignment error in the optical setup, authors in^17^ enhanced the proposed network of^15^ and replaced the strided convolution layer by a spectral pooling layer. Also, they replaced FC layer by a GAP operation to increase robustness of the system to the position variation of input images. Since they evaluated the architecture with simple MNIST^27^ and Fashion MNIST datasets, involvement of more complicated input images and their corresponding translations are missing in this study. Moreover, the lack of optical nonlinearity in the proposed network causes numerous electro-optical conversions in this architecture, which obviously limit the processing speed, as well as increase the power consumption overhead for large datasets processing.

With all this considered, although researchers have proposed few number of translation invariant optical neural networks, there is still a lack of all-optical and more general translation invariant architectures capable of addressing complicated datasets with large number of pixel shifts in all directions (i.e. horizontal, vertical, and diagonal). Proposing all-optical CNN with high level of translation invariance property not only helps the network to achieve higher classification accuracy, especially at the presence of alignment errors of optical components or translation of input images, but also leads to reduced power consumption at the absence of optoelectrical conversions in between layers. Therefore, in this paper, we propose an all-optical CNN which accurately classifies translated images in different directions. For this purpose, we enhance the translation invariance property of OP-AlexNet architecture^3^, and propose a new masking filter in 4f system to implement motion pooling operation. The main contributions of Trans-ONN are summarized as follows.

- Taking advantages of optical motion pooling with three different Gaussian masks in an all-optical convolutional layer based on wave optics-based code.
- Taking advantages of three different Gaussian masks (i.e. vertical, horizontal, and diagonal masks) as the convolution-based pooling filters for classifying translated test images toward the horizontal, vertical, and diagonal directions, respectively.
- Training and testing the proposed network on three different datasets of MNIST, CIFAR-10^28^, and Kaggle Cats and Dogs^29^ to obtain more generalized results.
- Achieving the maximum classification accuracy for the test images shifted by fewer than 24 pixel, while for larger shifts, Trans-ONN achieves higher classification accuracy, compared to the well-known AlexNet.

The rest of the paper is as follows. In section “Method and materials”, we discuss the AlexNet structure and explore the optical components as well as the adopted optical architecture (Trans-ONN). Section “Results” discusses the simulation scenarios and the results for three different datasets. In section “Comparison of trans-ONN against AlexNet”, we discuss classification accuracy improvement of Trans-ONN against AlexNet. Sections “Speed analysis” and “Power analysis” address the speedup and power consumption of Trans-ONN, respectively. Finally, Section “Conclusions” concludes the paper and presents the future works.

## Method and materials

CNNs as famous deep learning architectures are feedforward and sparsely connected networks which introduced in 1980^30^. The main building blocks of these networks include input layer, convolutional layers, nonlinearity layers, pooling layers, FC layers, and output layer^30^. In common CNN architectures, firstly, the input image is convolved with a set of trainable kernels in the convolutional layer. Secondly, the resultant feature maps pass through a nonlinear layer which performs a nonlinear activation function. As the next stage, in the pooling layer, an arithmetic process, such as subsampling operation, is applied on the input data fed by the nonlinear layer. These successive operations are performed in an iterative manner, and finally, in the FC layers, the incoming feature maps are multiplied by the trainable weight arrays. By applying softmax on the output values of the final FC layer, the probability distribution of each class is demonstrated and the classification accuracy is specified.

### Trans-ONN architecture

AlexNet, proposed by Alex Krizhevesky et al.^31^, is a CNN architecture that won the ImageNet Large Scale Visual Recognition Challenge (ILSVRC) in 2012^32^, at which it made a great influence on recognition accuracy against all the conventional machine learning as well as computer vision methods that specify it as a key achievement in object classification problems through the history^32^. The architecture of AlexNet consists of five convolutional layers and three FC layers. Convolution, ReLU, Local Response Normalization (LRN), and max pooling are the main building blocks of the first and the second convolutional layers of AlexNet, while the third and the fourth layers only contain the convolution and ReLU building blocks. Finally, the fifth layer consists of convolution, ReLU, and max pooling blocks^3^. Due to the fascination properties of optical processing which elaborated upon, the authors in^3^ proposed OP-AlexNet in which, the first layer of AlexNet is implemented optically while the subsequent layers are implemented electrically. They utilized optical 4f system to implement the convolution operation, optical saturable absorption as the optical nonlinear activation function, and finally, another optical 4f system, to implement the optical pooling operation.

Due to the misalignment errors in optical implementation, designing translation invariant optical CNN is of great interest and still is a challenging work. Although, the authors in^3^ introduced optical motion pooling to enhance the translation invariance property of OP-AlexNet, they used the translated images through the training process, which leads to acceptable accuracy for up to six pixel shifts in the translated test images.

**Figure 1:**
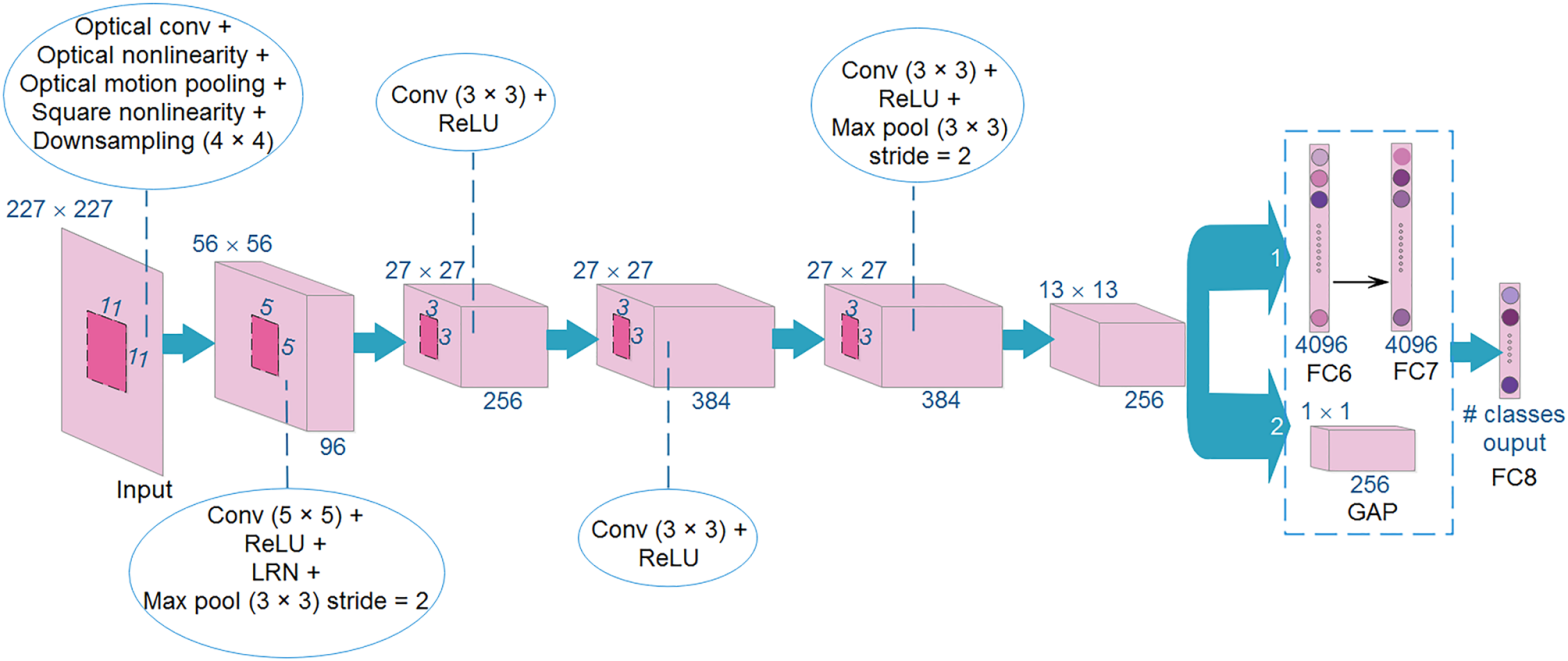
The schematic of Trans-ONN architecture.

To address the aforementioned drawbacks, in this work, we enhance the translation invariance property of OP-AlexNet architecture^3^. For this purpose, we design various masking filters to implement optical motion pooling operation to achieve high classification accuracy without the need of translated images through the training process. Moreover, we replace FC layers by GAP layer to reduce the sensitivity of FC layers’ feature maps to position variation of the input images. Figure 1 illustrates details of the proposed network, which named as Trans-ONN (i.e. optical convolutional neural network with enhanced translation invariance property). As shown in this figure, we compare two design alternatives: (a) using FC layers after the last convolutional layer, and (b) utilizing GAP layer at the end of last convolutional layer. The details of the adopted optical components are presented in the following sections.

### 4f system as optical Fourier convolution

One of the most common arithmetic operations in signal processing is the cross-correlation which measures the similarity between two signals or sequences. By considering the input signal as g(u,v) and the filter as s(u,v), the cross-correlation operation can be obtained in the frequency domain according to Eq. (1):

1 $$ c\left( {x,y} \right) = F\left\{ {G\left( {u,v} \right)S^{ * } \left( {u,v} \right)} \right\}, $$

where, $F$ is the Fourier transform function, G(u,v) is the Fourier transform of g(u,v), S^*^(u,v) is the complex conjugate of s(u,v), and finally, c(x,y) represents the 2D correlation of the two functions^33^. It is worth mentioning that correlation and convolution operations slightly differ in the time-domain, since according to the convolution operation, the signal is reversed prior to the multiplication process. Therefore, assuming symmetric filters, which is the case in CNNs, the outputs of convolution and cross-correlation operations would be similar.

**Figure 2:**
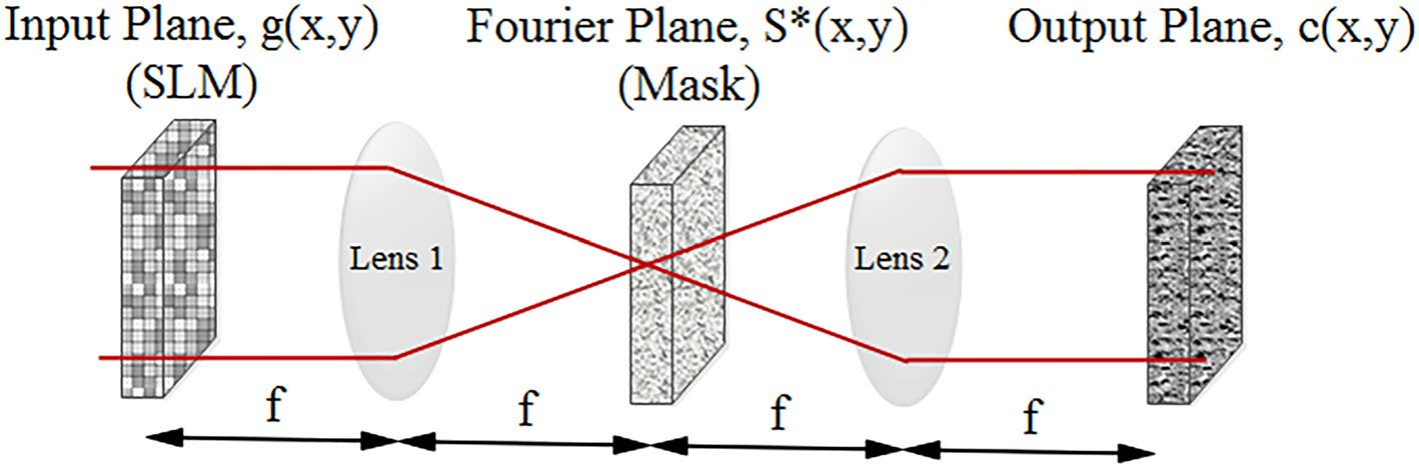
The schematic of 4f optical correlator.

Optical correlation can be realized by the famous 4f system in free-space optics which is based on the Vanderlugt setup^34^. The latter structure includes an input plane, two Fourier lenses each with the focal length of f next to the masking filter in the Fourier plane, and an output plane. The architecture of the 4f system is shown in Fig. 2.

As shown in Fig. 2, there are some steps to optically calculate the cross-correlation of two images (i.e. input and reference images); at first, the input image is produced by an SLM, at the next step, by propagating the light through the first lens (lens 1), the Fourier transform of the input image appears at the Fourier plane, where the mask representing Fourier transform of the reference image is located. In this manner, by propagating the light through the Fourier plane, the multiplication of two Fourier transformed images occurs optically. Afterwards, the second lens (lens 2) produces the inverse Fourier transform of the multiplication output, as the correlation of input and reference images. This final result either is detected by a photodetector for other electrical processing or propagates to the next step for further optical processing.

### Saturable absorption as optical nonlinear activation function

**Figure 3:**
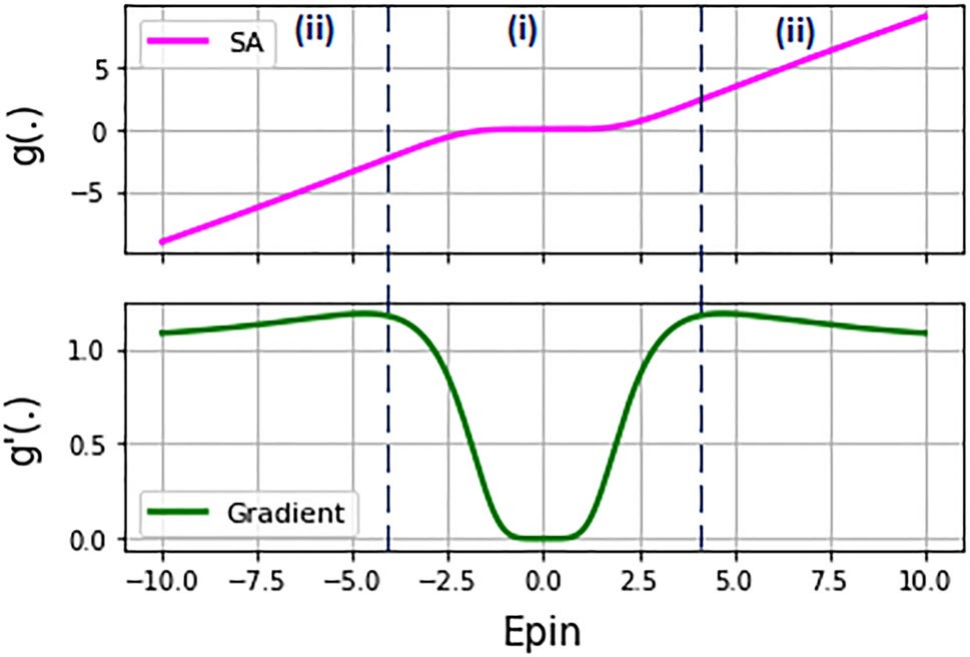
The schematic of SA nonlinearity.

Proposing an optical nonlinearity has been a debatable issue in recent years. So, as proposed in^3^, in this paper, we take advantages of a saturable absorber (SA) as the nonlinear layer, which is a fully-optical component based on an evacuated vapor cell filled with a gas-like rubidium atom^21^. This glass cell shows an absorption spectrum which causes a nonlinear input–output transform of the propagated light^21^. The mathematical model of the SA and its derivative curve are represented in Eqs. (2) and (3), respectively:

2 $$ E_{P,out} = g\left( {E_{P,in} } \right) = \exp \left( { - \frac{{{{\alpha_{0} } \mathord{\left/ {\vphantom {{\alpha_{0} } 2}} \right. \kern-\nulldelimiterspace} 2}}}{{1 + E_{P,in}^{2} }}} \right)E_{P,in} $$

3 $$ g^{\prime}\left( {E_{P,in} } \right) = \left[ {1 + \frac{{\alpha_{0} E_{P,in}^{2} }}{{\left( {1 + E_{P,in}^{2} } \right)^{2} }}} \right]\exp \left( { - \frac{{{{\alpha_{0} } \mathord{\left/ {\vphantom {{\alpha_{0} } 2}} \right. \kern-\nulldelimiterspace} 2}}}{{1 + E_{P,in}^{2} }}} \right) $$

where, E_P,in_ exhibits the input signal of SA, α_0_ is the resonant optical depth, E_P,out_ or g(E_P,in_) demonstrates the nonlinear output, and g′(E_P,in_) indicates the gradient output of SA. Figure 3 represents the input–output transfer function of SA, as well as its gradient assuming resonant optical depth of 20. As shown in this figure, the SA functionally behaves as a nonlinear unit in the specific region of input (i.e. region (i)), while in the other regions, it linearly transfers input signal to the output.

### Optical motion pooling layer

#### Pooling operation

As an inseparable component of the most CNNs, pooling operation can be considered as a subsampling or downsampling operation^35^. Furthermore, enjoying a broad spectrum of notable properties, including reducing overfitting as well as the computation time, and increasing recognition accuracy, to name but a few, the pooling operation is considered as the pivotal component in the visual recognition^35^. It should be noted that by performing the pooling operation, which shrinks the feature maps’ resolution, the irrelevant information of the feature maps, which is the result of convolution operation with nonlinear activation function, would be omitted, while preserving the main information. The latter property of pooling operation causes the network not being sensitive to the input deformations, and hence, results in a high classification accuracy^35^.

#### Translation invariance property

As many studies reveal^36–38^, the most usual pooling functions in CNNs are the max pooling and the average pooling^39^. Although these pooling operations obviously improve the network efficiency, they have not much effect on the translation invariance properties of CNNs. In a translation invariant neural network, the network’s outputs slightly change by applying translated input images^23^. Enhancing the translation invariance property of CNNs causes many benefits as: (1) fast and accurate target identification and tracking, specially in real-time applications^24^. The latter property raises from the fact that translation invariant CNNs do not require large datasets, with various shifted images. Therefore, classification process can be sped up^24^, (2) by enhancing the translation invariance properties of CNNs, translated images are not considered in the training process, and hence, training phase, as the most time consuming phase, can be sped up as well, and finally, (3) being translation invariant make optical CNNs more robust against misalignment problems which usually happen in the experimental setups.

#### Optical motion filters

Based on the above discussion, In this paper, we address the translation invariance property of the optical CNNs by generalizing the motion pooling layer of the predesigned OP-AlexNet architecture^3^. For this purpose, we propose utilization of vertical, horizontal, and diagonal (two-cascaded filters) optical motion filters. Based on their geometric design, optical motion filters blurs the image in a specific direction, which facilitates recognition of translated images and improves the classification accuracy^3^. A motion filter adds ghost-like artifacts to the image and makes the exact position of the object quite fuzzy. Training a model with this uncertainty in the objects’ position, within the input image, results in a translation invariance model.

**Figure 4:**
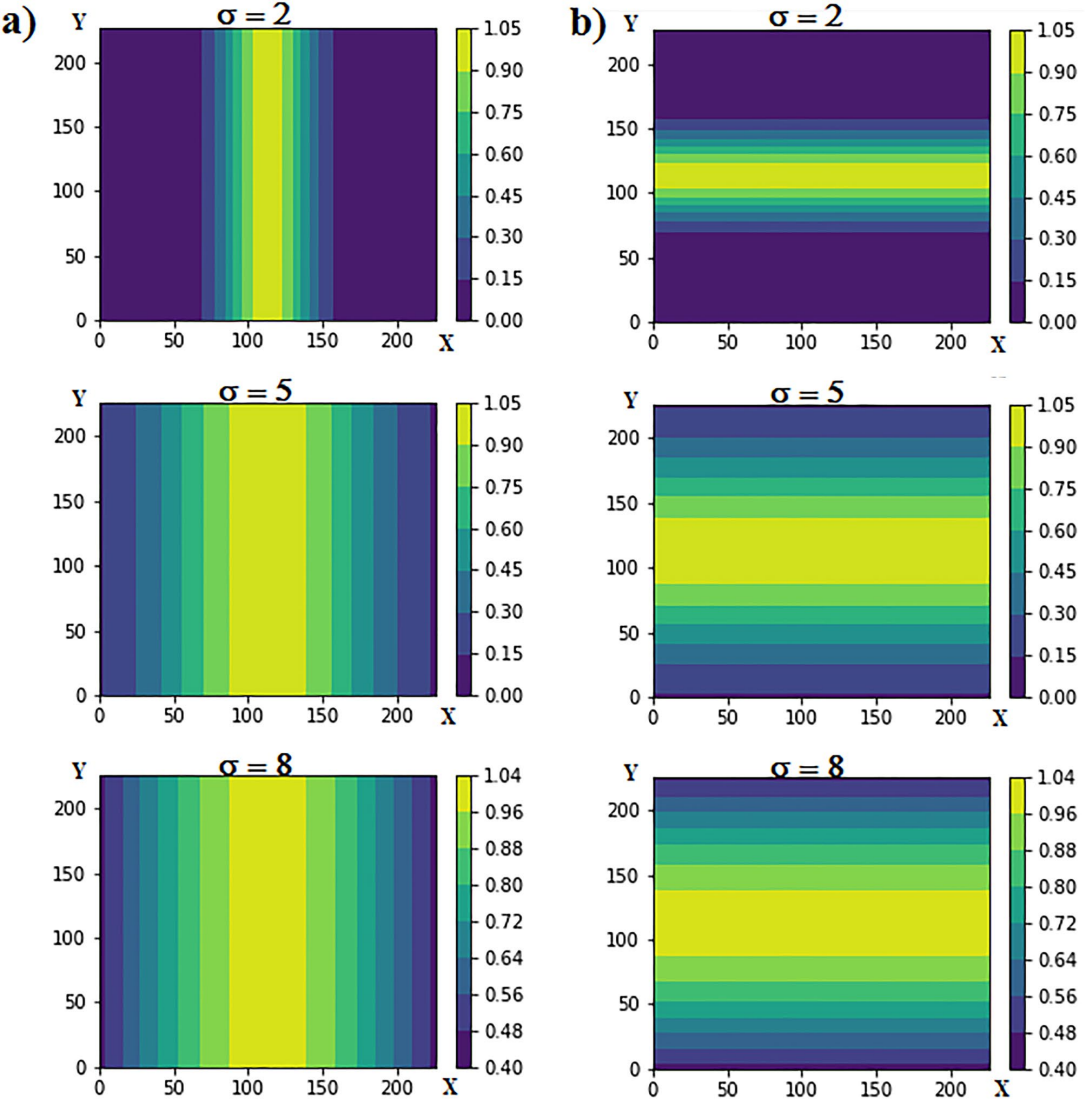
(**a**) Vertical motion filter and (**b**) horizontal motion filter each with w = 227.

Motion pooling can be realized in an optical neural network by a 4f system utilizing Gaussian mask in the Fourier plane, while adoption of wide Gaussian pattern leads to more motion blurring. To address translation invariance property in general, in this paper, three different motion filters, namely vertical, horizontal, and diagonal, are demonstrated as follows. Equations (4) and (5) represent the Gaussian mathematical model of vertical and horizontal motion filters, respectively. It is worth noting that the fabrication of these Gaussian masks can be easily achieved by printing the corresponding pattern on a piece of a transparent substrate^3^.

4 $$ Z_{v} \left( {x,y} \right) = \exp \left( {\frac{{ - \left( {x - {w \mathord{\left/ {\vphantom {w 2}} \right. \kern-\nulldelimiterspace} 2}} \right)^{2} }}{{2\sigma^{2} }}} \right) $$

5 $$ Z_{h} \left( {x,y} \right) = \exp \left( {\frac{{ - \left( {y - {w \mathord{\left/ {\vphantom {w 2}} \right. \kern-\nulldelimiterspace} 2}} \right)^{2} }}{{2\sigma^{2} }}} \right) $$

where, w is the mask’s width, $\sigma$ represents the standard deviation of the pattern by means of which the pattern’s width is measured, and finally, the intensity of pixel at point (x,y) is represented by Z(x,y). It should be noted that Z = 0 represents the black pixels blocking the light, and Z = 1 demonstrate the transparent pixels passing through the light^3^. For more clarity three samples of the vertical and horizontal motion filters are represented in Fig. 4.

**Figure 5:**
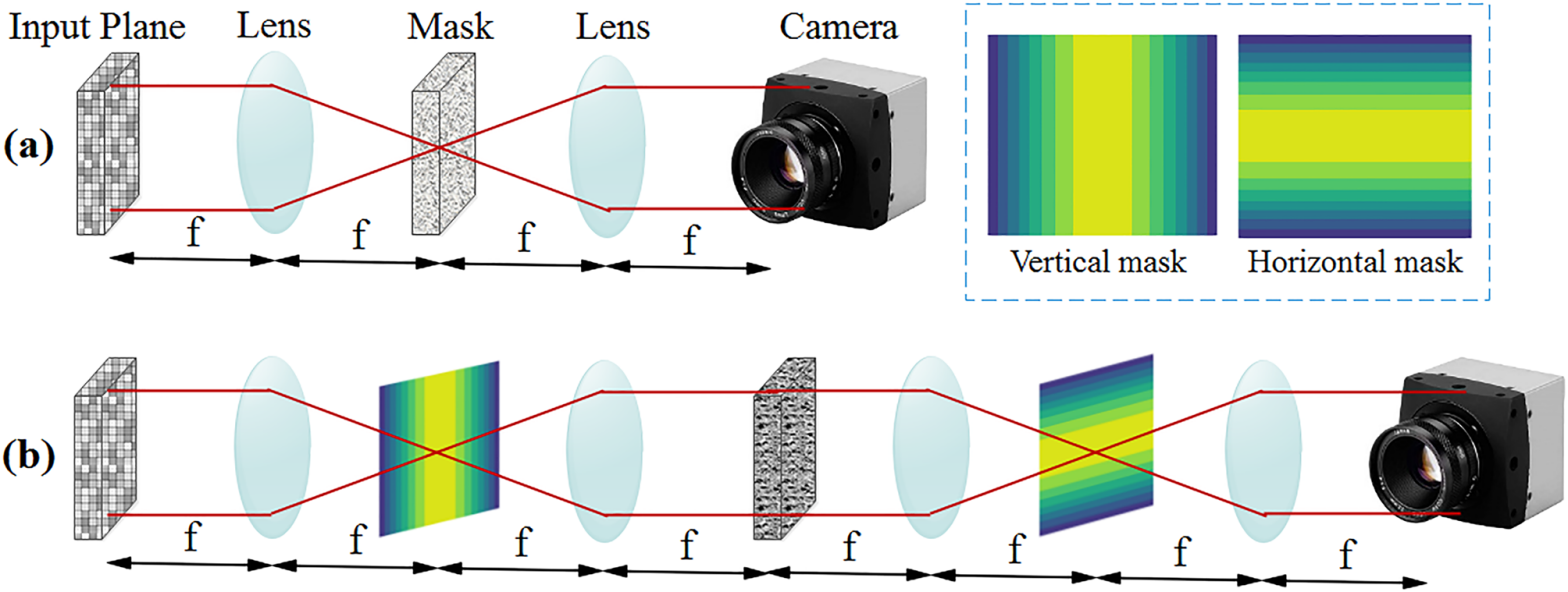
(**a**) 4f system implementing optical motion pooling in horizontal or vertical direction and (**b**) two cascaded 4f systems implementing an optical motion pooling in diagonal direction.

To enhance the classification accuracy of the translated images in a diagonal direction, we propose adoption of a diagonal mask which can be realized by two cascaded 4f systems in which the first one contains a vertical mask and the second one contains a horizontal mask in the Fourier plane. For more clarity, Fig. 5 shows different structures of motion pooling implementation in the proposed optical network, Trans-ONN.

**Figure 6:**
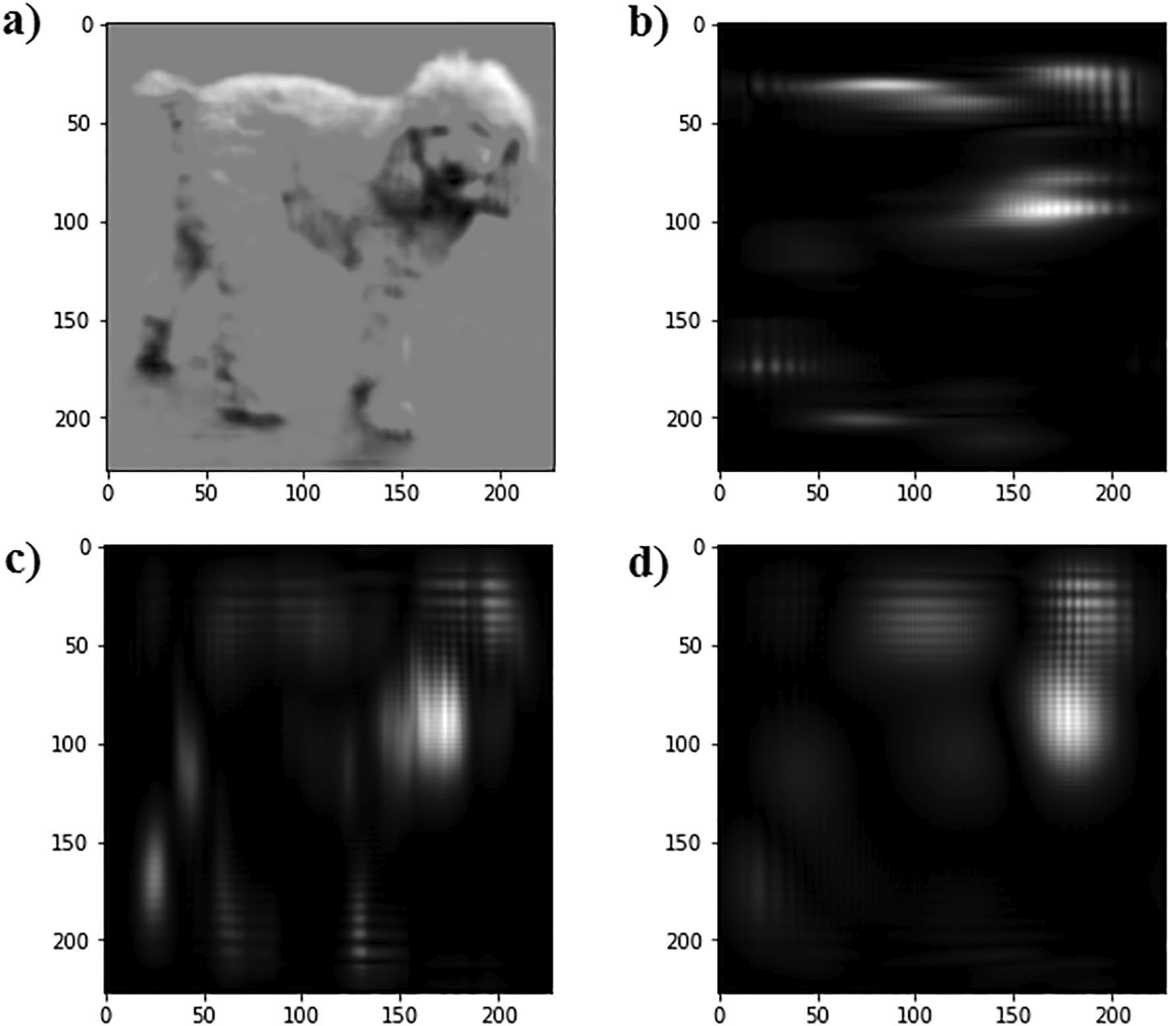
(**a**) input image, (**b**) correlation result of input image with vertical motion filter with w = 227 and $\upsigma$ = 2, (**c**) correlation result of input image with horizontal motion filter with w = 227 and $\upsigma$ = 2, and (**d**) correlation result of input image with diagonal motion filter with w = 227 and $\upsigma$ = 2.

Figure 6 represents an example of input image correlation with three different motion filters, implemented with vertical, horizontal, and diagonal masks. As shown in Fig. 6b,c, the vertical mask leads to motion blur in the horizontal direction, while the horizontal mask leads to motion blur in the vertical direction, respectively. Also the diagonal mask blurs the image in both directions (as shown in Fig. 6d).

### All-optical convolutional layer

**Figure 7:**
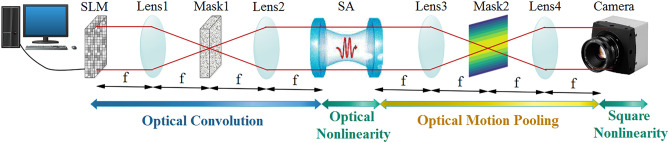
All-optical structure of an operation in first convolutional layer^3^.

To design an all-optical convolutional layer, we utilize stack of two lenslet arrays as the 4f optical correlators performing convolution operations with array of masks realizing different trainable kernels^3^ in the Fourier plane. Utilizing flat lenses, which is based on metasurfaces, the phase shift (φ_L_) of point (x,y) is represented by Eq. (6)^40^,

6 $$ \varphi_{L} = \frac{2\pi }{\lambda }\left( {\sqrt {x^{2} + y^{2} + f^{2} } - f} \right) $$

where, λ is the wavelength of the light, and f is the focal length of each lens. To design masking filters realizing positive and negative kernels in the optical setup, a checkerboard pattern of subpixels is proposed, as discussed in^3^. Once the convolution operations are accomplished, the resultant image propagates to the stack of array-based SA nonlinearities and the second 4f system implementing the optical motion pooling operations. Finally, the output image is captured by an array of detectors and is saved digitally for further electrical processing. It should be noted that the diameter and the focal lengths of lenses are considered 0.57 mm and 3 mm, respectively, which result in negligible crosstalk^3^. Also, a coherent light source with wavelength of 532 nm is assumed for generating the grayscale input image. Figure 7 shows the structure of an all-optical convolutional layer, as defined in^3^, by utilizing horizontal mask in the Fourier plane to implement optical motion pooling.

**Figure 8:**
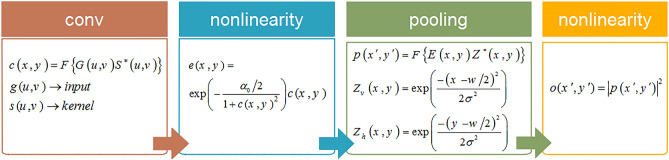
The mathematical function of each optical operation implemented in the proposed all-optical convolutional layer.

For more clarity, the mathematical function of each optical operation implemented by the Trans-ONN is represented in Fig. 8.

### GAP operation instead of FC layers

Although FC layers have considerable effect on the CNN’s performance, their functionality is dramatically influenced by the position variation of input images, as even a small shift of input data reduces accuracy of images classification^17^. The latter drawback is due to the fact that through the training process, the position of the extracted features, produced by the convolutional layers, and the weights array of FC layers, are significantly related to each other. In this manner, feeding translated images through the testing process results in undesired mismatches between the FC layers’ weights distribution and the extracted features at the convolutional layers’ output. In order to resolve this accuracy reduction, we applied GAP operation (by utilizing a customized tensorflow framework’s function) instead of FC6 and FC7 layers. In this manner, by global averaging of extracted features, the network can eliminate the aforementioned dependency problem and achieves accuracy improvement.

## Results

### Datasets

It is indubitably axiomatic that evaluation datasets are of pivotal importance in every simulation study. Here, we used three different datasets, namely Kaggle Cats and Dogs, CIFAR-10, and MNIST, to evaluate various simulation scenarios. The Kaggle Cats and Dogs includes 37.5 K RGB images of two classes of cat and dog images. The CIFAR-10 dataset contains 60 K 32 × 32 RGB images categorized to 10 classes, namely airplane, automobile, bird, cat, deer, dog, frog, horse, ship, and truck, each of which containing 6 K images. Finally, famous MNIST consists of 70 K 28 × 28 grayscale images of handwriting digits (0–9). It should be noted that based on AlexNet^31^ architecture, we resized all input images to 227 × 227 pixels. Also RGB images are converted to grayscale ones by a preprocessing operation. Table 1 represents the size of training, validation, and test images.

**Table 1:** Number of images for network training, validation and test.

Dataset	#Total images (K)	#Training images (K)	#Validation images (K)	#Test images (K)
Kaggle cats and dogs	37.5	30	2.5	5
CIFAR-10	60	45	5	10
MNIST	70	55	5	10

### Simulation and results

#### Network models

To evaluate Trans-ONN, different varieties of Trans-ONN network are proposed, the details of which are represented in Table 2.

**Table 2:** Different structures for implementing first convolutional layers.

Name	Convolution	Bias	Nonlinearity	Norm	Pooling	Nonlinearity	More info
AlexNet	Conv2D(11*11) and stride of 4	Bias	ReLU	LRN	Max-pool(3*3) and stride of 2	–	–
Trans-ONN-vert	Wave-optics Conv	–	SA	–	Wave-optics Motion-pool (by vertical mask)	Sqnl and DS(4*4)	–
Trans-ONN-vert-GAP	Wave-optics Conv	–	SA	–	Wave-optics Motion-pool (by vertical mask)	Sqnl and DS(4*4)	Considering GAP instead of FC6 and FC7
Trans-ONN-horiz	Wave-optics Conv	–	SA	–	Wave-optics Motion-pool (by horizontal mask)	Sqnl and DS(4*4)	–
Trans-ONN -horiz-GAP	Wave-optics Conv	–	SA	–	Wave-optics Motion-pool (by horizontal mask)	Sqnl and DS(4*4)	Considering GAP instead of FC6 and FC7
Trans-ONN -cascaded	Wave-optics Conv	–	SA	–	Wave-optics Motion-pool (2 cascaded 4f systems)	Sqnl and DS(4*4)	–
Trans-ONN -cascaded-GAP	Wave-optics Conv	–	SA	–	Wave-optics Motion-pool (2 cascaded 4f systems)	Sqnl and DS(4*4)	Considering GAP instead of FC6 and FC7

The details of each network are described as follow:

- **AlexNet** This network implement the AlexNet architecture proposed in^31^.
- **Trans-ONN-vert** In this network, some modifications on AlexNet are applied as follows: (1) we implemented 4f system by the wave optics-based code as the optical convolution operation, (2) for an easier implementation in the optical domain, we omitted bias term in the first convolutional layer of AlexNet^31^ architecture, (3) to implement an all-optical network, there is a need of optical nonlinearity unit. Therefore we utilized optical SA nonlinearity, instead of electrical ReLU operation, in the first layer of AlexNet^31^ architecture. Also performing input normalization to gather most SA’s input within the nonlinear region, we ignored LRN operation in the first layer structure, (4) as the main difference, we implemented 4f system by the wave optics-based code as the optical motion pooling operation and the proposed vertical mask are implemented by proper masks in the 4f system, and finally, (5) square nonlinearity (Sqnl) is applied as the photodetector‘s response function and a downsampling (DS) operation by factor of 16 is modeled after Sqnl.
- **Trans-ONN-vert-GAP** To evaluate the effect of GAP operation, we implemented Trans-ONN-vert-GAP by applying GAP operation instead of FC6 and FC7 layers in Trans-ONN-vert.
- **Trans-ONN-horiz** Considering the proposed horizontal mask instead of vertical mask in Trans-ONN-vert, the Trans-ONN-horiz is modeled to evaluate the impact of horizontal mask on the classification accuracy.
- **Trans-ONN-horiz-GAP** The Trans-ONN-horiz-GAP architecture is modeled by applying GAP operation instead of FC6 and FC7 in Trans-ONN-horiz to evaluate the effect of GAP operation in improving classification accuracy
- **Trans-ONN-cascaded** This network is implemented by implementing pooling operation by two cascaded 4f systems, rather than one in Trans-ONN-vert architecture, in which the first one contains a vertical mask and the second one contains a horizontal mask in the Fourier plane.
- **Trans-ONN-cascaded-GAP** Utilizing GAP operation, instead of FC6 and FC7 of Trans-ONN-cascaded, the Trans-ONN-cascaded-GAP is modeled.

#### Simulation environment

To simulate the aforementioned models, we extended the wave optics-based code of^41^ which is based on the Tensorflow-Python framework and utilizes the fast Fourier transform (FFT) algorithm, angular spectrum propagator, and complex-valued masks^6^. The developed simulator is executed on a GPU (NVIDIA GeForce GTX). Moreover, we applied the SA functionality as the optical nonlinearity, and so, to keep the convolutional features’ values within the nonlinear region of SA, we normalized all network models’ input values to the range of [− 5, 5]. On the other hand, we modeled the motion pooling layer by a wave optics-based code of the depth-wise convolution operation and we applied Gaussian filters as the Fourier plane masks. To obtain the maximum classification accuracy, we considered a Gaussian distribution function with a std value of, ${1 \mathord{\left/ {\vphantom {1 {\sqrt {fan_{in} } }}} \right. \kern-\nulldelimiterspace} {\sqrt {fan_{in} } }}$ for weight initialization of all convolutional layers, where fan_in_ equals k^2^c for a convolutional layer with k × k kernel size and $c$ number of input channels.

It should be noted that to obtain the optimized values of the networks parameters, several training simulations have been ran on the validation datasets, and finally, the best learning rate and the batch size have been chosen for each simulation model, whose details presented in section “Network parameters” of “Supplementary materials”. Moreover, we assumed that the training procedure is stopped once the rate of change of training accuracies and cross entropies reduces to less than 10^−3^.

#### Translation scenarios and classification accuracy

**Figure 9:**
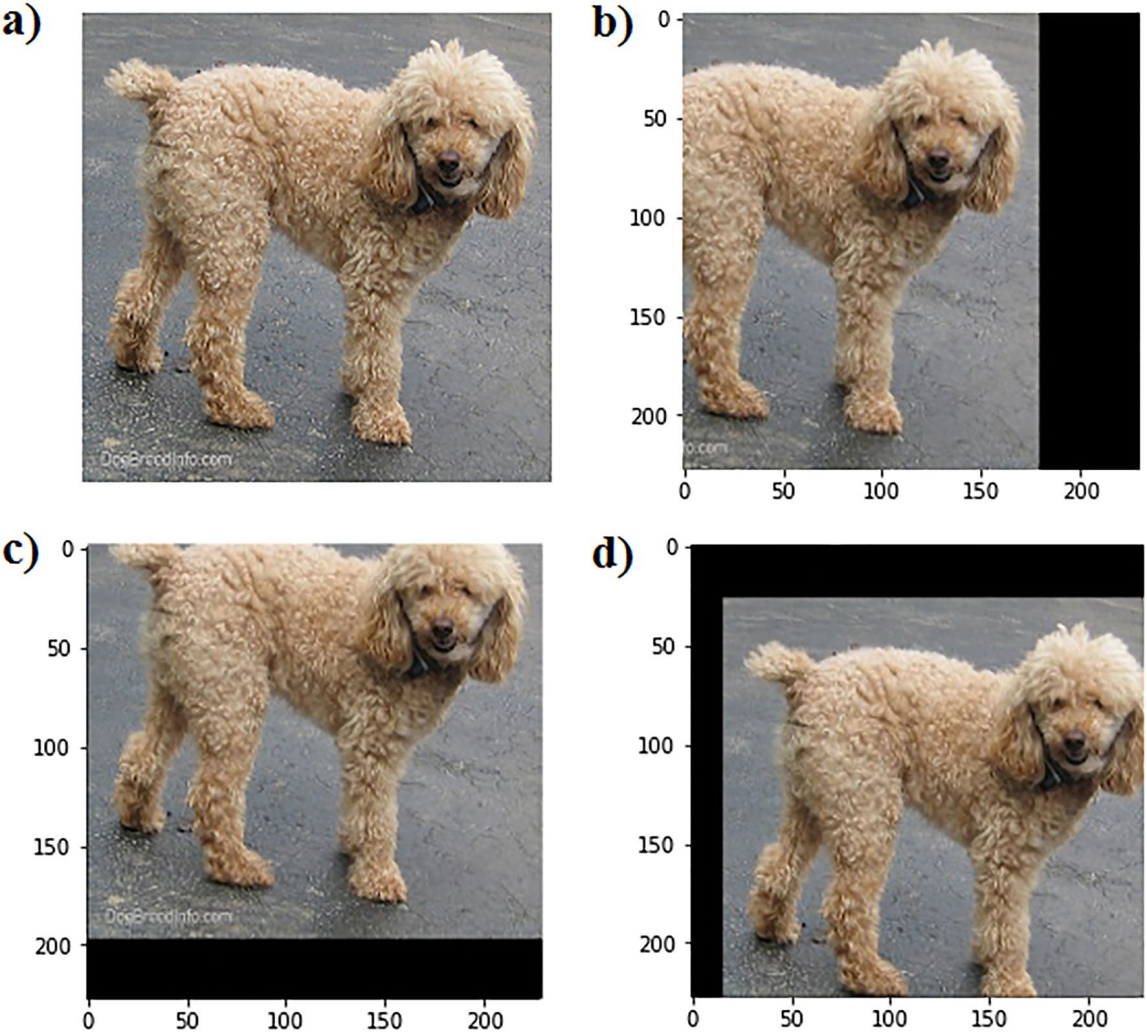
(**a**) A sample image from Kaggle Cats and Dogs dataset, and its translated version by (**b**) − 50 pixels shift toward the x-direction, (**c**) − 30 pixels shift toward the y-direction, and (**d**) (15, 26) pixels shift toward the (x,y) direction.

To evaluate the translation invariance capability of the networks, first we trained all networks by the original (i.e. non-shifted) training images, and then, we evaluated the networks considering three translation scenarios, where the test images were translated (1) up to 50 pixels in the x-direction, (2) up to 50 pixels in the y-direction, and (3) up to the 43 and 25 pixels toward the x and y direction, respectively. More details of the aforementioned translation scenarios are presented in Section “Details of various translation scenarios” of “Supplementary materials”. Furthermore, the translated test images are generated by random selection of 1.5 K images out of the 5 K Kaggle’s original test images, and 3 K images out of the 10 K for both CIFAR-10’s and MNIST’s original test images, while the aforementioned translated images were generated from each of these selected images. For more clarity, a sample image from Kaggle Cats and Dogs dataset and its sample translated images for the aforementioned scenarios are presented in Fig. 9.

As the ground truth, Table 3 represents the classification accuracy of all trained network models for original test images without involving any translated images.

**Table 3:** Classification accuracy of original test images for different simulation models.

Model’s name/dataset	Kaggle Cats and Dogs	CIFAR-10	MNIST
AlexNet	88.52	78.50	99.12
Trans-ONN-vert	82.12	70.21	99.35
Trans-ONN-vert-GAP	83.16	69.69	98.78
Trans-ONN-horiz	83.44	72.11	99.31
Trans-ONN-horiz-GAP	85.14	69.79	98.63
Trans-ONN-cascaded	80.34	67.43	98.77
Trans-ONN-cascaded-GAP	77.2	63.53	98.92

**Figure 10:**
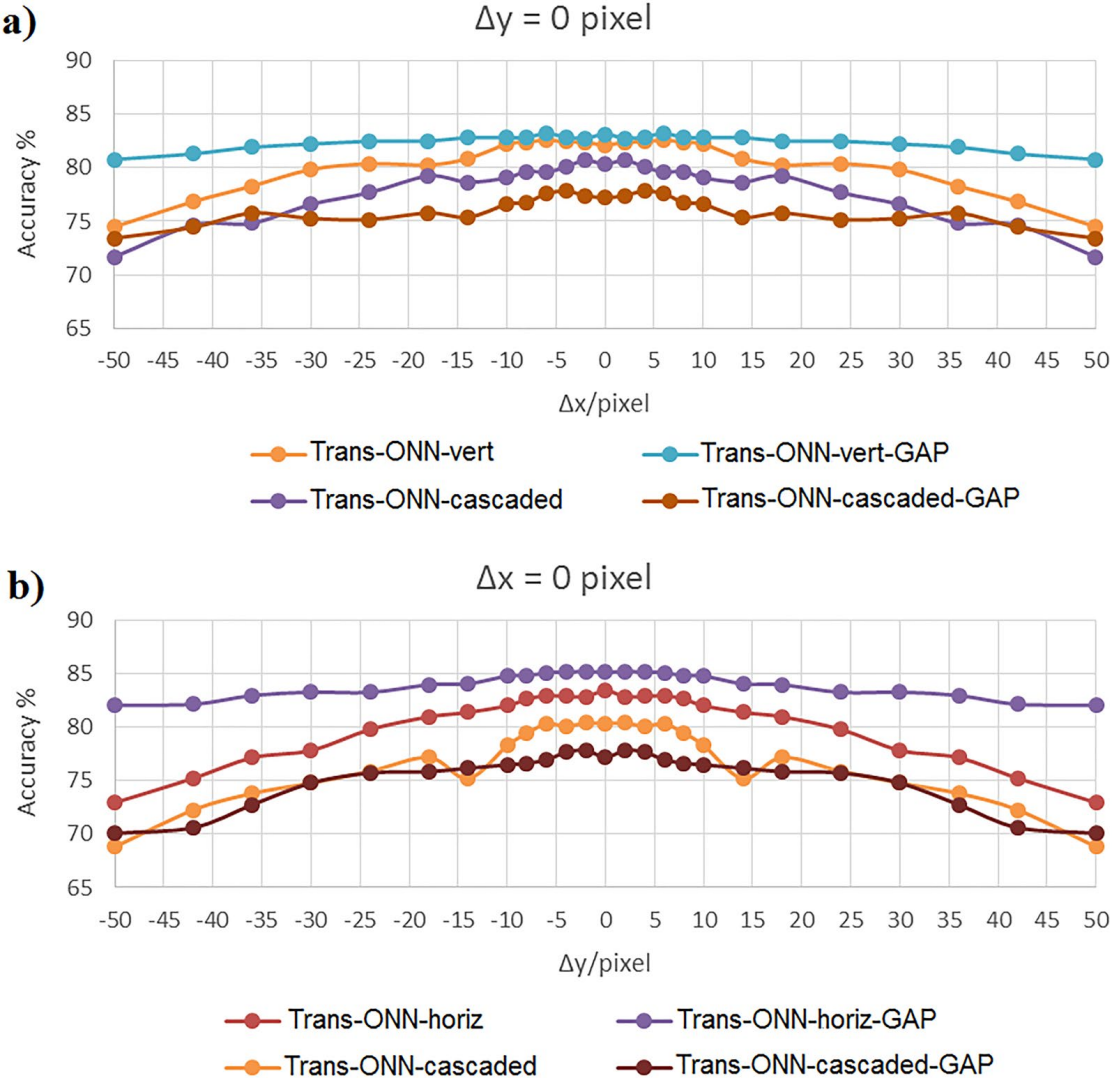
Classification accuracy of Kaggle Cats and Dogs dataset by considering (**a**) vertical mask, and (**b**) horizontal mask.

**Figure 11:**
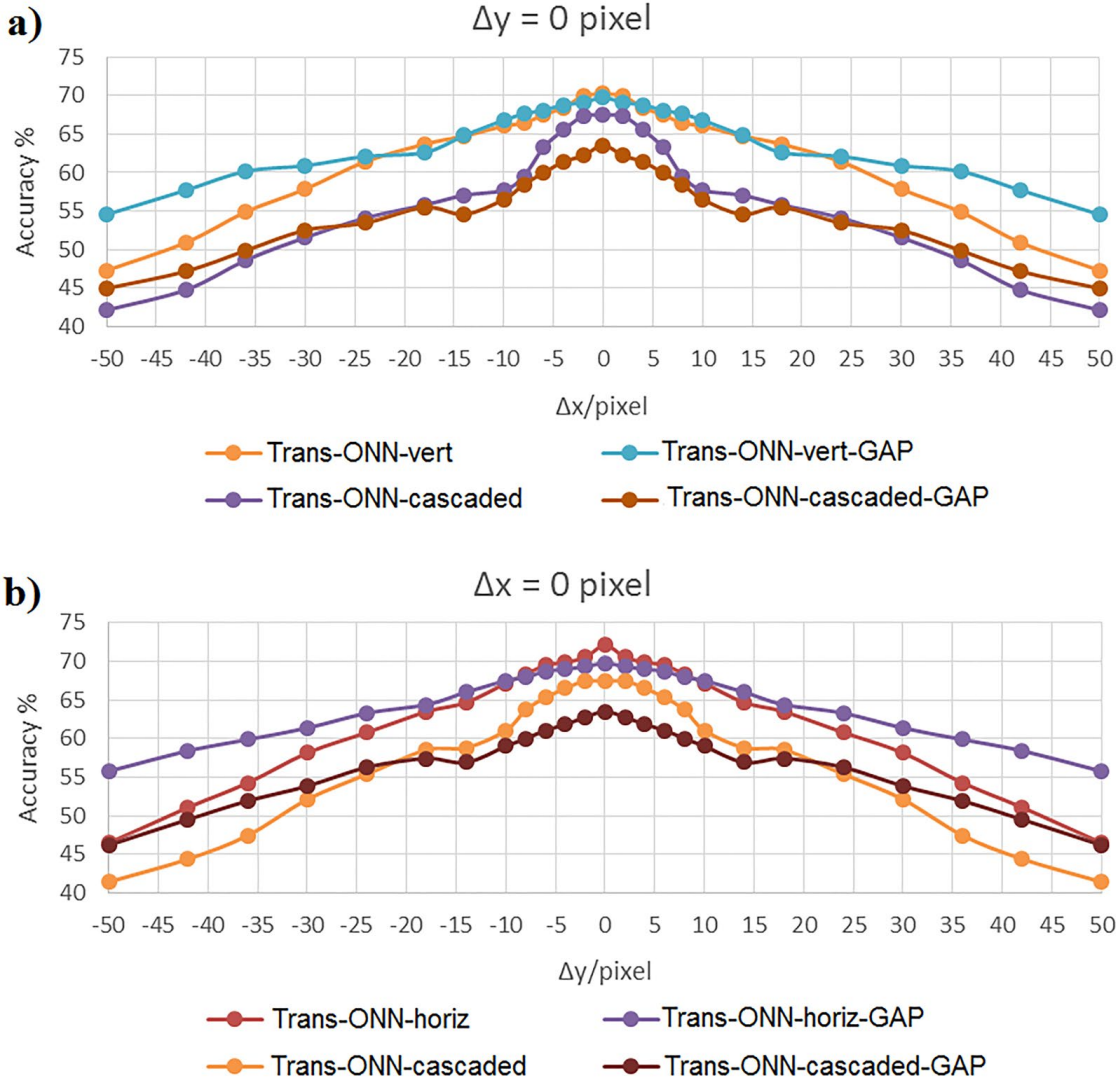
Classification accuracy of CIFAR-10 dataset by considering (**a**) vertical mask, and (**b**) horizontal mask.

**Figure 12:**
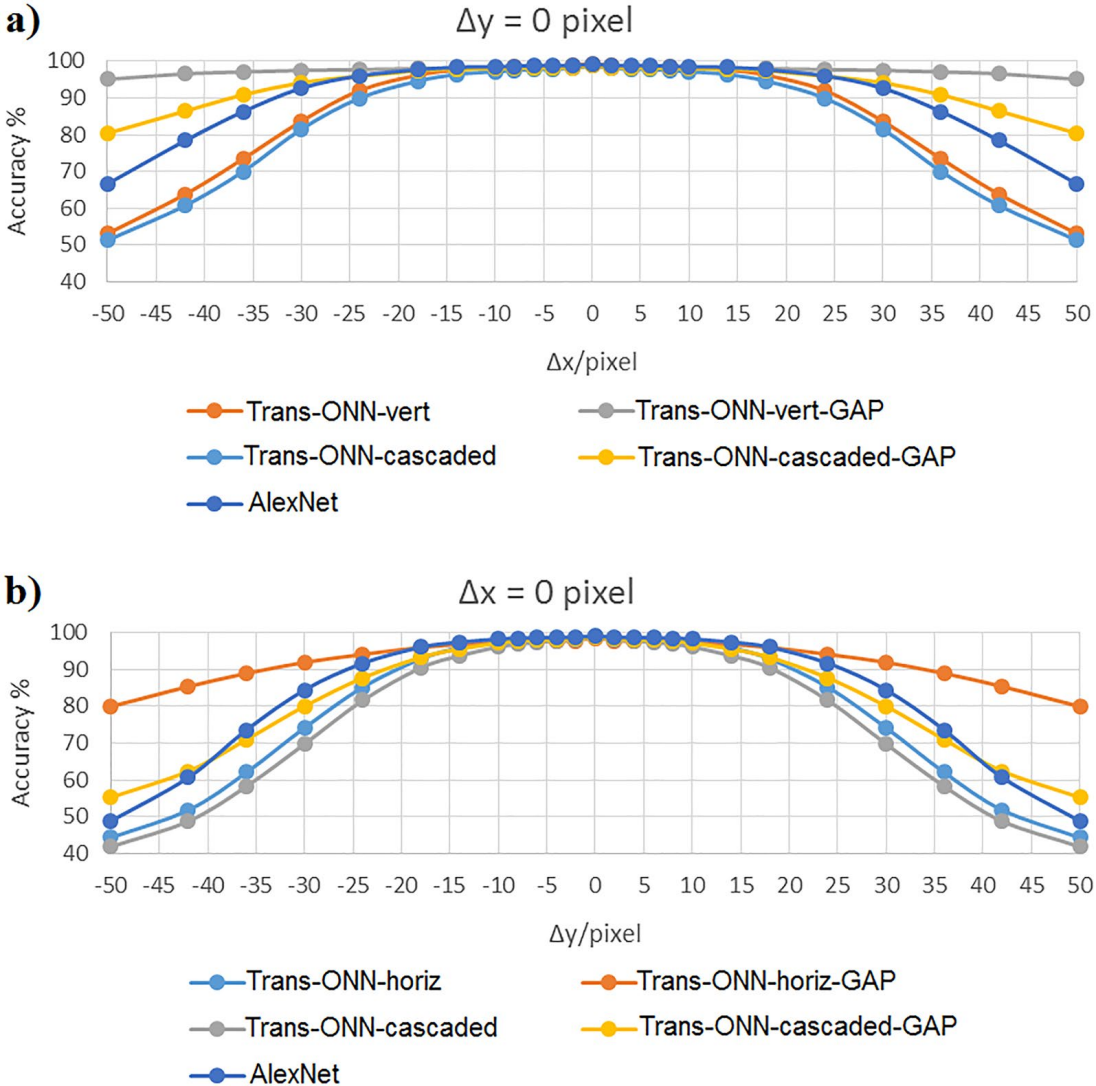
Classification accuracy of MNIST dataset by considering (**a**) vertical mask, and (**b**) horizontal mask.

Figures 10, 11, and 12 show the achieved classification accuracies for the first and second translation scenarios, in each (a) and (b) represent the first and the second translation scenario, respectively. These figures illustrate the accuracy sensitivity of different network models to the image translation in the x- and y-direction, for Kaggle Cats and Dogs, CIFAR-10, and MNIST datasets, respectively. It is worth mentioning that the negative and positive numbers in the x-direction represent the number of pixel shifts to the left and right, respectively, while the negative and positive numbers in the y-direction represent the number of pixel shifts upward and downward, respectively. It should be noted that although the training procedure is based on the original test images, both the original test images and their translated versions are involved in the testing procedure.

As shown in Fig. 10a, for Kaggle Cats and Dogs dataset and considering the first translation scenario, Trans-ONN-vert-GAP network model provides the best translation invariance property Specifically, it can classify the translated test images, generated by horizontal shifts of − 30, − 24, − 18, − 14, − 10, − 8, − 6, − 4, − 2, 2, 4, 6, 8, 10, 14, 18, 24, and 30 pixels, with nearly the same accuracy achieved by the ground truth model (second column, third row of Table 3). Also, for horizontal shifts of test images by more than − 30 and 30 pixels, the Trans-ONN-vert-GAP experiences a slight accuracy reduction, unlike the alternative network models. On the other hand, as shown in Fig. 10b, for the second translation scenario, the Trans-ONN-horiz-GAP achieves the best translation invariance property as well. Specifically, it can classify the translated test images, generated by vertical shifts of − 14, − 10, − 8, − 6, − 4, − 2, 2, 4, 6, 8, 10, and 14 pixels, with nearly the same accuracy achieved by the ground truth model (second column, fifth row of Table 3). Also, for vertical shifts of test images by more than − 14 and 14 pixels, the Trans-ONN-horiz-GAP leads to a slight accuracy reduction, unlike the alternative network models.

As shown in Fig. 11a,b, for CIFAR-10 dataset, Trans-ONN-vert-GAP and Trans-ONN-horiz-GAP achieve the best translation invariance property, compared to the alternative network models, for the first and the second translation scenarios, respectively. Specifically, both networks can classify the translated test images, generated by − 6, − 4, − 2, 2, 4, and 6 pixel shifts with nearly the same accuracy achieved by the ground truth model (third column, third and fifth rows of Table 3). Also, for horizontal or vertical shifts of test images by more than − 6 and 6 pixels, the both network models leads to a slight accuracy reduction, unlike the alternative network models.

As shown in Fig. 12a,b, for MNIST dataset, Trans-ONN-vert-GAP and Trans-ONN-horiz-GAP achieve the best translation invariance property, compared to the alternative network models, for the first and the second translation scenarios, respectively. Specifically, the Trans-ONN-vert-GAP can classify translated test images generated by − 36, − 30, − 24, − 18, − 14, − 10, − 8, − 6, − 4, − 2, 2, 4, 6, 8, 10, 14, 18, 24, 30, and 36 pixel shifts with nearly the same accuracy achieved by the ground truth model (forth column, third row of Table 3). The Trans-ONN-horiz-GAP can classify translated test images generated by − 10, − 8, − 6, − 4, − 2, 2, 4, 6, 8, and 10 pixel shifts with nearly the same accuracy achieved by the ground truth model (forth column, fifth row of Table 3). Also, for horizontal shifts of test images by more than − 36 and 36 pixels, the Trans-ONN-vert-GAP network model leads to a slight accuracy reduction, unlike the alternative network models. Moreover, for vertical shifts of the test images by more than − 10 and 10 pixels, the Trans-ONN-horiz-GAP network model leads to a slight accuracy reduction, unlike the alternative network models.

**Figure 13:**
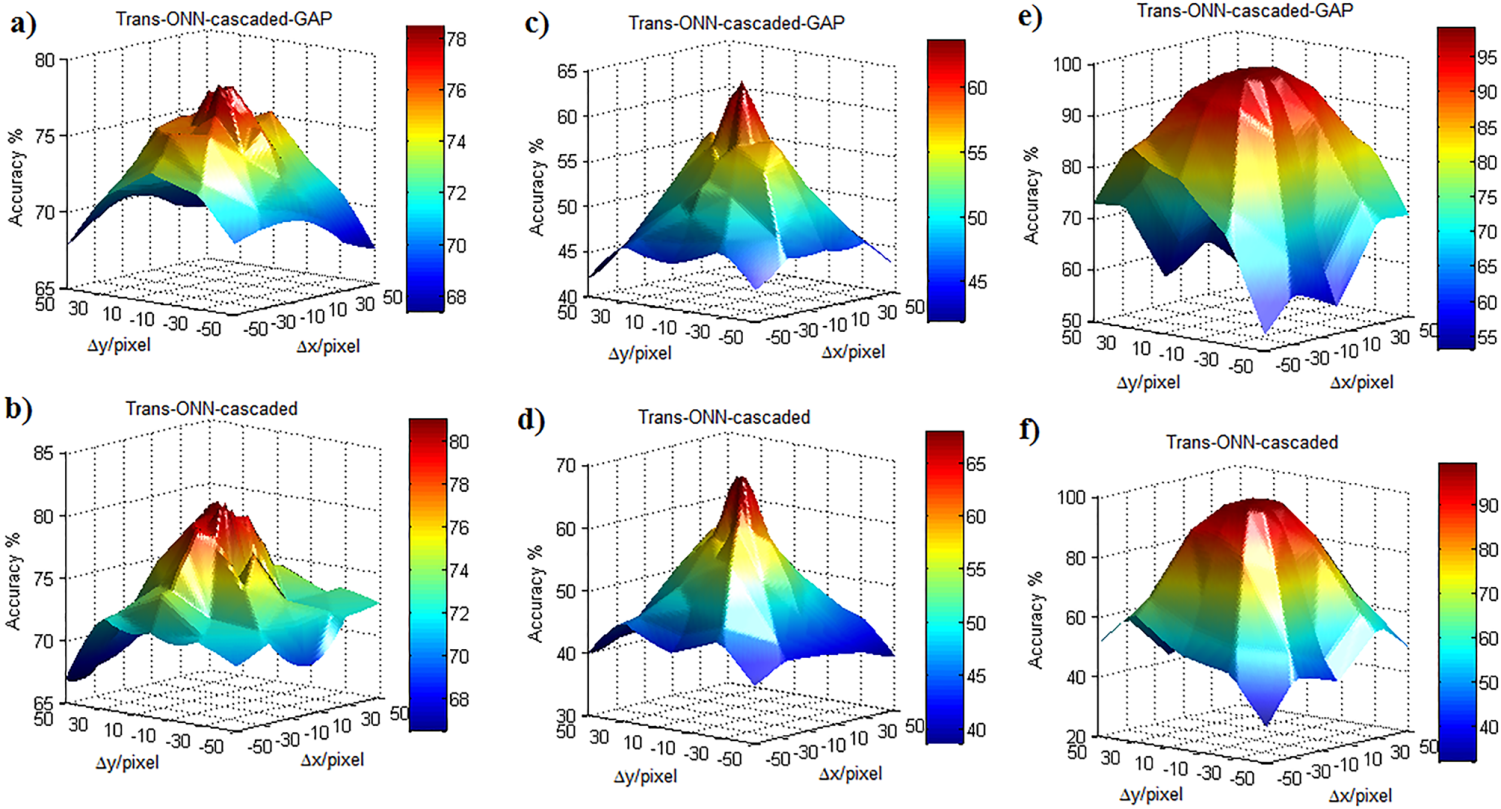
Classification accuracy of translated test images in both x and y directions, for (**a**) Trans-ONN-cascaded-GAP for Kaggle Cats and Dogs dataset, (**b**) Trans-ONN-cascaded networks for Kaggle Cats and Dogs dataset, (**c**) Trans-ONN-cascaded-GAP for CIFAR-10 dataset, (**d**) Trans-ONN-cascaded networks for CIFAR-10 dataset, (**e**) Trans-ONN-cascaded-GAP for MNIST dataset, (**f**) Trans-ONN-cascaded networks for MNIST dataset.

To evaluate the impact of adopting the diagonal mask, the third translation scenario is considered, where the test images are translated toward both x and y directions. Figure 13 shows the achieved classification accuracies for this scenario for Kaggle Cats and Dogs, CIFAR-10, and MNIST datasets, respectively. The classification accuracies for various number of pixel shifts of the third scenario are explored in Section “Simulation results of adopting diagonal mask” of “Supplementary materials”. Considering Table 3, the ground truth accuracy of Trans-ONN-cascaded is higher than that of Trans-ONN-cascaded-GAP for both Kaggle Cats and Dogs and CIFAR-10 datasets, while for MNIST dataset both simulation models have the same accuracies. In this manner, as shown in Fig. 13a–d, the maximum accuracy of Trans-ONN-cascaded is slightly higher than that of Trans-ONN-cascaded-GAP, but the point is that the rate of accuracy reduction, by increasing the number of pixel shifts, is much smaller for Trans-ONN-cascaded-GAP, compared to the Trans-ONN-cascaded model for both Kaggle Cats and Dogs and CIFAR-10 datasets. Furthermore, Trans-ONN-cascaded-GAP achieves better classification accuracy, compared to Trans-ONN-cascaded for more than 30 pixel shifts, which can be noted as the significant impact of GAP adoption in Trans-ONN-cascaded-GAP. As shown in Fig. 13e,f, for MNIST datasets, for any number of pixel shifts, the Trans-ONN-cascaded-GAP achieves higher classification accuracy, compared to Trans-ONN-cascaded, with the accuracy difference of more than 10% for large number of pixels shifts.

To compare AlexNet with Trans-ONN, as an example we included the classification accuracies of AlexNet in Fig. 12 for MNIST dataset. As shown in this figure, the classification accuracies of AlexNet fed by different translated test images of MNIST dataset demonstrate is less than those of Trans-ONN-vert-GAP and Trans-ONN-horiz-GAP for classifying horizontally and vertically shifted test images by more than 24 pixel shifts, respectively. Specifically, as shown in Table 4, the standard deviation (std) values of MNIST classification accuracies achieved by Trans-ONN-vert-GAP and Trans-ONN-horiz-GAP are 0.91 and 5.69 for classifying horizontally and vertically shifted test images, respectively, while utilizing AlexNet, we can achieve the std values of 9.98 and 16.59 for classifying horizontally and vertically shifted test images. These results shows the valuable translation invariance property of Trans-ONN (Trans-ONN-vert-GAP and Trans-ONN-horiz-GAP) providing higher classification accuracies over AlexNet for classifying translated test images produced by large pixel shifts (i.e. more than 24 pixel shifts).

**Table 4:** Standard deviation of classification accuracies for each network model.

Model’s name/dataset	Kaggle Cats and Dogs	CIFAR-10	MNIST
AlexNet (horizontally shifted test images)	–	–	9.98
AlexNet (vertically shifted test images)	–	–	16.59
Trans-ONN-vert	2.49	7.18	15.36
Trans-ONN-vert-GAP	0.7	4.66	0.91
Trans-ONN-horiz	3.29	7.92	19.44
Trans-ONN-horiz-GAP	1.12	4.51	5.69
Trans-ONN-cascaded	3.28	8.29	17.22
Trans-ONN-cascaded-GAP	2.25	5.51	10.99

As shown in Table 4, the std value of Trans-ONN-vert-GAP and Trans-ONN-horiz-GAP are much lower than those of other network models for all three datasets which demonstrate the best translation invariance properties of these two Trans-ONN network models.

In summary, having considered all the aforementioned simulation scenarios, we can conclude that the Trans-ONN-vert-GAP and Trans-ONN-horiz-GAP provide the best translation invariance property, compared the all alternative network models, for classifying horizontally and vertically shifted test images, respectively. On the other hand, by adopting the diagonal masks, the rate of accuracy reduction, by increasing the number of pixel shifts, is much smaller for Trans-ONN-cascaded-GAP, compared to the Trans-ONN-cascaded model for both Kaggle Cats and Dogs and CIFAR-10 datasets. Also, Trans-ONN-cascaded-GAP achieves higher classification accuracy, compared to Trans-ONN-cascaded for large number of pixel shifts (i.e. more than 30 pixel shifts). It is worth mentioning that, as the key advantages of the optical motion pooling layer, the proposed translation invariant networks are capable of classifying the translated test images not included in the training procedure.

### Comparison of trans-ONN against OP-AlexNet

As mentioned before, the Trans-ONN is considerably improved over OP-AlexNet^3^, by including translation invariant pooling layer. To clarify this improvement, the classification accuracy of Trans-ONN is compared with that of OP-AlexNet at the presence of translated test images as follows. It should be emphasized that the OP-AlexNet^3^, in general, is not translation invariant, although it was evaluated by the horizontally shifted MNIST images. Specifically, for evaluating the OP-AlexNet, the test images are translated toward the x-direction, and then, resized to 227 × 227 pixels. With this in mind, to not losing the main structure of the handwriting digits of MNIST dataset, maximum horizontal shift of 6 pixels is considered for evaluating OP-AlexNet. However, for the case of Trans-ONN network, firstly, the input images are resized to 227 × 227 pixels, and then, the resized images are translated to generate the test images.

For a fair comparison, as shown in Table 5, we compare the classification accuracy of OP-AlexNet with that of Trans-ONN by considering translated test images generated by horizontal shift of 4, 6, − 4, and − 6 pixels (maximum pixels shift in the OP-AlexNet). It should be noted that the OP-AlexNet is evaluated in two scenarios; first, the translated images are included in the training procedure, and second, the translated images are not considered through the training process. In this regard, we can compare accuracy of the OP-AlexNet with that of the Trans-ONN for both scenarios, as shown in Table 5. Considering the first and the second rows of Table 5, the OP-AlexNet leads to classification accuracies of 97.69% and 61.90% with and without including the translated images in the training process, respectively. On the other hand, as shown in third row of Table 5, due to the translation invariance properties of Trans-ONN-vert-GAP structure (Trans-ONN considering vertical mask in motion pooling and GAP operation), it achieves the classification accuracy of 98.13%, which is improved compared to the OP-AlexNet. It is worth noting that the latter classification accuracy of Trans-ONN is achieved without inclusion of translated images in the training procedure, which confirms its outperformance at the presence of alignment noises.

**Table 5:** Comparison of OP-AlexNet against Trans-ONN.

	Network	Train dataset	Test dataset	# Pixels shift in each image of translated train images	# Pixels shift in each image of translated test images	Accuracy (%)
1	OP-AlexNet	Original + translated images	Original + translated images	3, 5, − 3, − 5	4, 6, − 4, − 6	97.69
2	OP-AlexNet	Original images	Original + translated images	–	4, 6, − 4, − 6	61.90
3	Trans-ONN-vert-GAP	Original images	Original + translated images	–	4, 6, − 4, − 6	98.13

### Speed analysis

**Figure 14:**
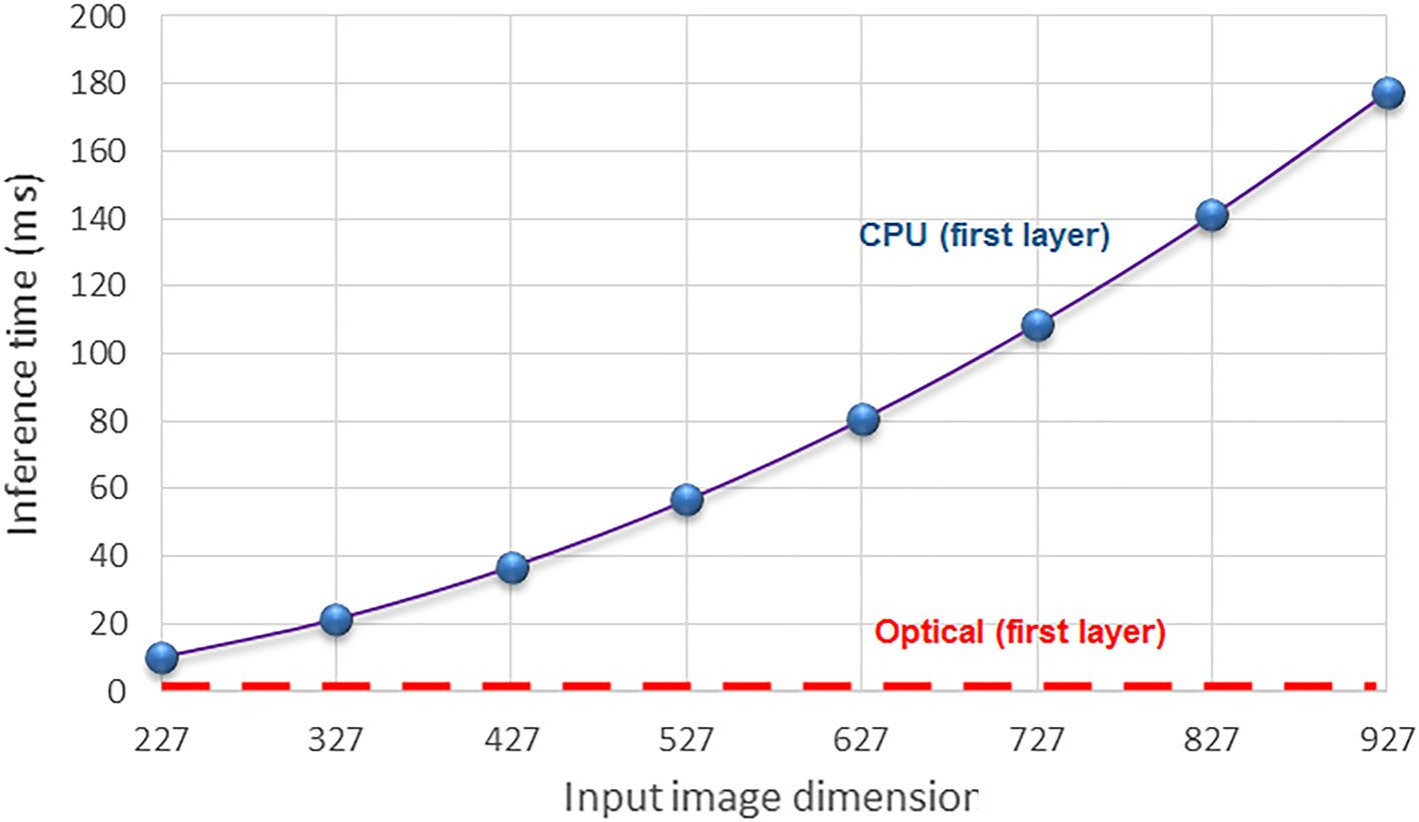
Inference time of the optical and electrical implementations of the first layer of AlexNet in terms of input image dimension.

It is undeniable that the ultimate goal of proposing an all-optical CNN is increasing the speed of processing, and so, avoiding the high latency of electrical implementations. To estimate the speedup achievable by the Trans-ONN, the latency of its optical layer is calculated as follows:

7 $$ Latency = T_{source} + T_{4f\_conv} + T_{SA} + T_{4f\_pool} + T_{camera} + T_{transfer\_data} $$

where, Latency demonstrates latency of the first optical layer, T_source_ represents delay of the input image generation which depends on SLM’s switching rate. Considering switching frequency of 1 kHz for the SLMs^6^, T_source_ would be 1 ms. T_4f_conv_ and T_4f_pool_ represent the optical propagation delays through the convolution and pooling operations, respectively. Considering 4f. optical correlators, T_4f_conv_ and T_4f_pool_ approximately equal 10 ps^3,6^. T_SA_ represents the latency of SA nonlinearity which is 25 ps^3^. T_camera_ stand for the latency of photodetectors to capture output images and convert them to the electrical data. Taking advantages of high-speed commercial cameras^42^, at the speed of 2500 frames per second, the latency of the camera can be measured as 0.4 ms. Finally, T_transfer_data_ is the latency of the communication interface to transmit camera’s output to a computer. By considering USB 3.1 Gen2 with a frame rate of 10 Gbit/s and assuming 100 kB image, T_transfer_data_ would be equal to 0.08 ms. To sum it up, latency of the first optical layer of Trans-ONN using horizontal, vertical, or diagonal mask, as the pooling layer, is estimated as 1.48 ms, while inference computation time of the first layer of AlexNet^31^ is about 10.15 ms by assuming an input image of size 227 × 227 pixels and CPU processor frequency of 3.8 GHz (Intel Core i7 8 core, Skylake-X microarchitecture). More details on measuring electrical computation time of each convolutional layer are explored in Section “Inference runtime estimation” of “Supplementary materials”. Summarizing above discussion, we achieve speedup of 6.86, compared to the AlexNet, for the first convolutional layer. Furthermore, as shown in Fig. 14, by increasing the input image size, latency of the electrical convolutional layer increases linearly by the input image dimension, while latency of the proposed optical layer equals 1.48 ms and is independent of the input image dimension, for image sizes up to 3840 × 2160 pixels, which is the size of a 4 K UHD SLMs^43^.

### Power analysis

One of the noteworthy advantages of the optical neural network is its lower energy consumption, compared to the electrical counterpart. The energy required by the Trans-ONN is negligible thanks to the optical convolution, nonlinearity, and pooling operations^3^ and it depends on the signal transduction. Assuming power consumption of 1 μW for capturing each pixel at the detector, power consumption of an optical network can be measured by^3,6^:

8 $$ P_{optical} = \frac{{n^{2} \times n_{\ker nel} }}{{\eta \times t^{p} }}\mu W, $$

where, n^2^ is the total number of pixels per 4f. correlator, p represents the number of optical elements through the path, n_kernel_ is the number of different kernels of the convolutional layer, t is a fraction of incident power each optical element transmits, and finally, η is the source efficiency.

The total energy required by the electrical implementation is measured as^3,6^:

9 $$ P_{electronic} = \beta \times n^{2} \times k^{2} \times n_{\ker nel} \times P_{switching} , $$

where the constant coefficient β is determined by the executed architecture, k^2^ is the kernels’ size and P_switching_ is the specific amount of energy consumed by each operation.

It is worth mentioning that although the energy consumption of both optical and electrical convolutional layers linearly increases with the number of pixels and the number of kernels, kernel size does not impact energy consumption of the optical setup. Thus, without any shadow of doubt, power consumption of the optically implemented convolutional layers can be greatly reduced, compared to the electrical one for the large kernel sizes.

## Conclusions

One of the fascinating solutions to overcome the alignment noises in the optical experimental setup is the adoption of optical translation invariant networks. In this regard, this paper proposes a free-space all-optical translation invariant CNN which is named Trans-ONN. The proposed optical CNN accurately classifies translated test images generated by various pixel shifts (up to 50 pixel shifts) in different directions of horizontal, vertical, and diagonal. Trans-ONN takes advantages of an optical motion pooling layer which provides the translation invariance property by implementing different optical masks in the Fourier plane for classifying translated test images of Kaggle Cats and Dogs, CIFAR-10, and MNIST datasets in all three directions. Moreover, to improve the translation invariance property, GAP operation is utilized in the Trans-ONN structure, rather than the fully connected layers, which improves the network classification accuracy for all three datasets. Specifically, taking advantage of the vertical and horizontal masks along GAP operation provide the best translation invariance property, compared to the alternative network models, for classifying horizontally and vertically shifted test images of Kaggle Cats and Dogs, CIFAR-10, and MNIST datasets. Also, adopting the diagonal mask along GAP operation, we can achieve the best classification accuracy for classifying translated test images in the diagonal direction for large number of pixel shifts (i.e. more than 30 pixel shifts). Finally, it is worth noting that the proposed architecture mitigates the rate of accuracy reduction, by increasing the number of pixel shifts, for Kaggle Cats and Dogs and CIFAR-10 datasets. As the future works, optical implementation of the GAP and FC layers would be addressed to realize a fully optical design of CNNs to achieve the maximum speedup and power reduction.

## Supplementary Information

Supplementary Information.

## Data Availability

Data underlying the results presented in this paper are available in Refs.^27–29^.
