# Supplementary material for: Translation-invariant optical neural network for image classification
Source: Sci Rep. 2022 Oct 14;12:17232. doi: 10.1038/s41598-022-22291-0 (PMC9568607; doi:10.1038/s41598-022-22291-0)
Supplement: Supplementary file 1 — Supplementary Information. [file 41598_2022_22291_MOESM1_ESM.pdf]

Supplementary Information: Translation-invariant optical neural network for image classification

Hoda Sadeghzadeh[1] & Somayyeh Koohi[1,*]

[1]Department of Computer Engineering, Sharif University of Technology, Tehran, Iran

[*]Correspondence: E-mail: koohi@sharif.edu

Network parameters

The best learning rate and the batch size for each simulation model are reported in Table S1.

Table S1. Learning Rate and Batch Sizes for Different Simulation Models

Name	Learning rate	Batch size
AlexNet	0.0001	16
Trans-ONN-vert	0.001	4
Trans-ONN-vert-GAP	0.001	4
Trans-ONN-horiz	0.001	4
Trans-ONN-horiz-GAP	0.001	4
Trans-ONN-cascaded	0.001	2
Trans-ONN-cascaded-GAP	0.001	2

Details of various translation scenarios

The number of pixel shifts in (x,y) direction for each translation scenario is represented in Table S2. In the x-direction, the negative and positive numbers represent the number of pixel shifts to the left and right, respectively, while the negative and positive numbers for the y-direction represent the number of pixel shifts upward and downward, respectively.

Table S2. Three Translation Scenarios

First scenario (x,y)	(-50,0), (-42,0), (-36,0), (-30,0), (-24,0), (-18,0), (-14,0), (-10,0), (-8,0), (-6,0), (-4,0), (-2,0), (2,0), (4,0), (6,0), (8,0), (10,0), (14,0), (18,0), (24,0), (30,0), (36,0), (42,0), (50,0)
Second scenario (x,y)	(0,-50), (0,-42), (0,-36), (0,-30), (0,-24), (0,-18), (0,-14), (0,-10), (0,-8), (0,-6), (0,-4), (0,-2), (0,2), (0,4), (0,6), (0,8), (0,10), (0,14), (0,18), (0,24), (0,30), (0,36), (0,42), (0,50)
Third scenario (x,y)	(1,-2), (-1,-2), (-1,2), (1,2), (2,-1), (-2,-1), (-2,1), (2,1), (3,-5), (-3,-5), (-3,5), (3,5), (5,-3), (-5,-3), (-5,3), (5,3), (7,-12), (-7,-12), (-7,12), (7,12), (12,-7), (-12,-7), (-12,7), (12,7), (15,-26), (-15,-26), (-15,26), (15,26), (26,-15), (-26,-15), (-26,15), (26,15), (25,-43), (-25,-43), (-25,43), (25,43), (43,-25), (-43,-25), (-43,25), (43,25)

Simulation results of adopting diagonal mask

Table S3, Table S4, and Table S5 show the classification accuracies of adopting diagonal mask considering Trans-ONN-cascaded-GAP and Trans-ONN-cascaded models with translated input images generated by various pixel shifts for Kaggle Cats and Dogs, CIFAR-10 and MNIST datasets, respectively.

Table S3. Test accuracy (%) considering original test images with their translated ones for Kaggle Cats and Dogs dataset

# pixel shifts (x,y)	Classification accuracy (%) of Trans-ONN-cascaded-GAP	Classification accuracy (%) of Trans-ONN-cascaded
(1,-2)	78.13	79.90
(-1,-2)	78.10	80.47
(1,2)	77.67	80.43
(2,-1)	78.13	80.17
(-2,-1)	77.93	80.53
(-2,1)	77.30	80.63
(2,1)	77.73	80.40
(3,-5)	77.47	79.43
(-3,-5)	77.77	79.57
(-3,5)	77.40	80.67
(3,5)	77.17	80
(5,-3)	78.10	79.50
(-5,-3)	78.40	80.57
(-5,3)	77.93	80
(5,3)	77.53	79.73
(7,-12)	76.57	73.83
(-7,-12)	77.23	74.50
(-7,12)	75.60	79.03
(7,12)	75.67	78.23
(12,-7)	75.33	77.80
(-12,-7)	76.60	78.50
(-12,7)	75.60	79.30
(12,7)	75.50	79.23
(15,-26)	73.03	73.10
(-15,-26)	74.37	75
(-15,26)	75.23	75.83
(15,26)	73.80	76.97
(26,-15)	73.87	72.47
(-26,-15)	75.70	72.57
(-26,15)	75.43	74.57
(26,15)	74.93	77.43
(25,-43)	69.50	72.37
(-25,-43)	71.30	72.17
(-25,43)	70.73	69.60
(25,43)	70.23	72.50
(43,-25)	71.57	73.07
(-43,-25)	73.13	71.90
(-43,25)	72.27	72.60
(43,25)	72.50	73.20

Table S4. Test accuracy (%) considering original test images with their translated ones for CIFAR-10 dataset

# pixel shifts (x,y)	Classification accuracy (%) of Trans-ONN-cascaded-GAP	Classification accuracy (%) of Trans-ONN-cascaded
(1,-2)	62.63	67.63
(-1,-2)	62.72	67.88
(1,2)	62.80	66.70
(2,-1)	62.17	67.12
(-2,-1)	62.53	67.73
(-2,1)	62.47	67.15
(2,1)	62.33	66.80
(3,-5)	60.92	65.30
(-3,-5)	61.58	66.08
(-3,5)	61.57	65.58
(3,5)	61.17	64.50
(5,-3)	60.33	64.13
(-5,-3)	61.63	65.37
(-5,3)	61.40	64.98
(5,3)	60.20	63.33
(7,-12)	56.85	57.78
(-7,-12)	57.27	60.35
(-7,12)	56.43	56.42
(7,12)	55.63	56.08
(12,-7)	55.42	57.12
(-12,-7)	57.17	60.80
(-12,7)	56.87	58.65
(12,7)	55.37	56.77
(15,-26)	52.20	50.08
(-15,-26)	54.80	52.95
(-15,26)	52.80	52.83
(15,26)	51.27	51.48
(26,-15)	51.58	51.12
(-26,-15)	52.37	55.85
(-26,15)	50.50	51
(26,15)	51.13	48.52
(25,-43)	47.52	42.92
(-25,-43)	48.23	44.70
(-25,43)	46.43	44.10
(25,43)	47.10	44.62
(43,-25)	46.87	45.58
(-43,-25)	48.20	47.33
(-43,25)	47.02	46.62
(43,25)	46.47	45.60

Table S5. Test accuracy (%) considering original test images with their translated ones for MNIST dataset

# pixel shifts (x,y)	Classification accuracy (%) of Trans-ONN-cascaded-GAP	Classification accuracy (%) of Trans-ONN-cascaded
(1,-2)	98.42	98.13
(-1,-2)	98.47	98.17
(1,2)	98.23	97.93
(2,-1)	98.28	98.15
(-2,-1)	98.52	98.15
(-2,1)	98.32	98.10
(2,1)	98.18	97.92
(3,-5)	98.52	97.97
(-3,-5)	98.42	98.10
(-3,5)	98.17	97.62
(3,5)	97.95	97.28
(5,-3)	98.43	97.92
(-5,-3)	98.48	98.18
(-5,3)	98.40	97.97
(5,3)	98.07	97.32
(7,-12)	97.75	96.27
(-7,-12)	97.72	96.62
(-7,12)	96.88	95.68
(7,12)	95.52	92.95
(12,-7)	97.88	96.42
(-12,-7)	98.30	97.38
(-12,7)	97.85	97.12
(12,7)	96.77	94.45
(15,-26)	89.63	82.70
(-15,-26)	88.67	79.60
(-15,26)	90.45	82.07
(15,26)	80	67.35
(26,-15)	93.88	84.70
(-26,-15)	93.37	83.28
(-26,15)	95.13	89.47
(26,15)	86.28	71.55
(25,-43)	74.35	61.92
(-25,-43)	66.78	53.78
(-25,43)	75.48	62.17
(25,43)	65.15	50.98
(43,-25)	83.82	64.18
(-43,-25)	75.85	56.12
(-43,25)	86.58	68.10
(43,25)	72.52	53.17

Inference runtime estimation

To investigate the impact of optical processing on reducing the execution time, compared to the electrical counterpart, we compare processing time of an optical convolutional layer with that of the electrical layer within a CNN. For the electrical network, the runtime estimation of each operational layer is estimated as follows:

$$\begin{aligned} T_{conv+relu} &= \left(\left(k_L^2 \times n_{ch} + 1\right) \times n_{kernel}\right) \times load + \left(k_L^2 \times n_{ch} \times n_{kernel}\right) \times N_{feature}^2 \times fmult + \\ &\left(\left(k_L^2 - 1\right) \times n_{ch} \times n_{kernel} + n_{ch} \times n_{kernel}\right) \times N_{feature}^2 \times fadd + \left(\left(n_{ch} + 1\right) \times n_{kernel}\right) \times N_{feature}^2 \times store + \\ &n_{kernel} \times N_{feature}^2 \times max + n_{kernel} \times N_{feature}^2 \times store \end{aligned} \quad \text{(S1)}$$

$$T_{lrn} = \left(n_{kernel} \times N^2_{feature}\right) \times load + \left(\left(n_{kernel} + 1\right) \times N^2_{feature}\right) \times fmult + \left(n_{kernel} \times N^2_{feature}\right) \times fadd + \left(n_{kernel} \times N^2_{feature}\right) \times power + \left(n_{kernel} \times N^2_{feature}\right) \times fdiv + \left(n_{kernel} \times N^2_{feature}\right) \times store \quad \text{(S2)}$$

$$T_{Maxpool} = \left(n_{kernel} \times N^2_{feature}\right) \times load + \left(n_{kernel} \times M^2_{feature} \times k^2_m\right) \times max + \left(n_{kernel} \times M^2_{feature}\right) \times store \quad \text{(S3)}$$

where, $T_{conv+relu}$ is the execution time of the convolution operations followed by a Relu, as the nonlinear activation function included in all five convolutional layer of AlexNet, k^2_L is the kernel size of the convolution operation, n_{ch} is the number of input channels for the convolutional layer, n_{kernel} is the number of kernels in each convolutional layer, and $N^2_{feature}$ is the size of convolutional output's feature maps. T_{lrn} is the execution time of the local response normalization (lrn) operation, which is utilized in the first and the second convolutional layers of AlexNet. $T_{Maxpool}$ is the execution time of the max pooling operation which is utilized in the first, second, and fifth layers of the AlexNet network architecture, $M^2_{feature}$ is the size of max pooling layer's feature maps, and k^2_m is the filter size of the max pooling operation. Finally, load, fmult, fadd, store, max, power, and fdiv present the required CPU clock latency (for Intel Core i7 8 core, Skylake-X microarchitecture) for executing the corresponding instructions, as shown in Table S6 [1] . It should be noted that except load and store operations, the remaining operations are executed in a parallel manner whose execution time are divided by the number of cores.

Table S6. Number of clock of each instruction [1]

Instruction	Latency
Load/store	1
fmult	5
fadd	3
Max	3
Power	14-21
fdiv	14-16

It should be noted that $N_{feature}$ as the dimension of the output matrix of the convolution operation is calculated as follows:

$$N_{feature} = \left\lfloor \frac{n_{in} + 2p - k_L}{s_{conv}} + 1 \right\rfloor \qquad \text{(S4)}$$

where n_{in} is the dimensions of the input matrix of the convolution operation, s_{conv} is the stride value of the convolution operation, and p is the padding size whose value is zero for VALID type, while for the SAME type it is considered as follow:

$$p = \frac{k_L - 1}{2} \qquad \text{(S5)}$$

Also $M_{feature}$ as the dimension of the output matrix of the max pooling operation is calculated as follow:

$$M_{feature} = \left\lfloor \frac{N_{feature} - k_m}{s_m} + 1 \right\rfloor \qquad \text{(S6)}$$

where, s_m is the stride value of the max pooling operation.

References

[1] Fog, A. Introduction 4. Instruction tables. https://www.agner.org/optimize/instruction_tables.pdf (1996).
